# Dyslexia Candidate Gene and Ciliary Gene Expression Dynamics During Human Neuronal Differentiation

**DOI:** 10.1007/s12035-020-01905-6

**Published:** 2020-05-22

**Authors:** Andrea Bieder, Masahito Yoshihara, Shintaro Katayama, Kaarel Krjutškov, Anna Falk, Juha Kere, Isabel Tapia-Páez

**Affiliations:** 1 Department of Biosciences and Nutrition, Karolinska Institutet, Hälsovägen 9, 141 57 Huddinge, Sweden; 2 Competence Centre on Health Technologies, Tartu, Estonia; 3 Research Program of Molecular Neurology, Research Programs Unit, University of Helsinki, Helsinki, Finland; 4 Folkhälsan Institute of Genetics, Helsinki, Finland; 5 Department of Neuroscience, Karolinska Institutet, Stockholm, Sweden; 6 School of Basic and Medical Biosciences, King’s College London, London, UK; 7 Department of Medicine, Solna, Karolinska Institutet, Solna, Sweden

**Keywords:** Cilia, Ciliopathies, Reading disorder, Human neuroepithelial stem cells, RNA sequencing

## Abstract

Developmental dyslexia (DD) is a neurodevelopmental condition with complex genetic mechanisms. A number of candidate genes have been identified, some of which are linked to neuronal development and migration and to ciliary functions. However, expression and regulation of these genes in human brain development and neuronal differentiation remain uncharted. Here, we used human long-term self-renewing neuroepithelial stem (lt-NES, here termed NES) cells derived from human induced pluripotent stem cells to study neuronal differentiation in vitro. We characterized gene expression changes during differentiation by using RNA sequencing and validated dynamics for selected genes by qRT-PCR. Interestingly, we found that genes related to cilia were significantly enriched among upregulated genes during differentiation, including genes linked to ciliopathies with neurodevelopmental phenotypes. We confirmed the presence of primary cilia throughout neuronal differentiation. Focusing on dyslexia candidate genes, 33 out of 50 DD candidate genes were detected in NES cells by RNA sequencing, and seven candidate genes were upregulated during differentiation to neurons, including *DYX1C1 (DNAAF4)*, a highly replicated DD candidate gene. Our results suggest a role of ciliary genes in differentiating neuronal cells and show that NES cells provide a relevant human neuronal model to study ciliary and DD candidate genes.

**Electronic supplementary material:**

The online version of this article (10.1007/s12035-020-01905-6) contains supplementary material, which is available to authorized users.

## Introduction

Developmental dyslexia (DD) is a learning disorder with a neurodevelopmental origin. It is the most common learning disorder present in about 5–10% of the population. Early studies in postmortem human brains have suggested underlying neuronal migration anomalies [1]. DD is highly heritable and many candidate genes have been proposed [2] (Suppl. Table 1). Some of the most genetically replicated DD candidate genes (DCGs), namely *DYX1C1*, *DCDC2*, and *KIAA0319*, have been implied in neuronal development and migration in rodents, supporting the early studies in human brains [3–5]. Interestingly, the same genes have been shown independently to have a role in ciliary biology [6–11]. Cilia are present on mammalian neuronal cells at different stages of development—in progenitor cells and post-mitotic neurons [12]. At present, little is known about their function in neuronal development and homeostasis, and their roles in neuronal proliferation, migration, and maturation are just beginning to be elucidated [13]. Many ciliopathies show neurologic symptoms, and cilia have been linked to neuropsychiatric disorders [14–18].

In contrast to the vast literature on genetic studies of DCGs, few functional studies have been carried out. Some studies in animal model systems and cell lines have addressed the regulation and functions of *DYX1C1*, *DCDC2*, and *KIAA0319* (reviewed in [19]). However, their function in human neuronal cells and cilia is still unsettled. While studies in model systems provide valuable insights, given the human-specific phenotype of DD, it is important to address DCG regulation in a human neuronal system. However, a systematic assessment of DCGs in human neuronal development is so far lacking. To study human-specific gene regulatory events and neurodevelopmental disorders, modeling human brain development in vitro derived from induced pluripotent stem cells (iPSCs)/embryonic stem cells (ESCs) combined with transcriptomic characterization has become a crucial tool [20]. Human long-term self-renewing neuroepithelial stem (lt-NES, here termed NES) cells derived from human iPSCs (hiPSCs) can mimic human neuronal development in vitro. They resemble neuroepithelial cells in vivo, self-renew in the presence of fibroblast growth factor (FGF) and epidermal growth factor (EGF), and can differentiate into neuronal and glial cells [21–23]. They have been used as a model for neurodevelopmental processes and disorders [24, 25].

Here, we sought to map gene expression changes during early human neuronal development in vitro with a focus on DCG regulation. We monitored gene expression throughout differentiation from NES cells to neuronal cells by RNA-sequencing (RNA-seq) on bulk RNA samples. In addition, we characterized specifically the dynamics of DCG expression.

## Materials and Methods

### Cell Culture

The ethical guidelines for derivation of cell line AF22 were described previously [21]. Reprogramming of human cells was permitted by Regional ethical committee Stockholm (Dnr 2012/208–31/3). The derivation and culturing of NES cells (line AF22, derived from a healthy female person) were described previously [21, 25]. Briefly, NES cells were cultured in DMEM/F12+Glutamax supplemented with penicillin (100 U/ml), streptomycin (100 μg/ml), N2 (1:100), B27 (1:1000), FGF (10 ng/ml) (all from Life Technologies, Thermo Fisher Scientific, Carlsbad, CA, USA), and EGF (10 ng/ml; Peprotech, Rocky Hill, NJ, USA) in a 5% CO_2_ incubator. Half of the medium was changed daily, and cells were passaged at a ratio of 1:3 upon confluency. Plates were pre-coated using poly-ornithine (0.1 mg/ml; Sigma-Aldrich, St. Louis, MO, USA) and laminin (2 μg/ml; Sigma-Aldrich, L2020). For differentiation, cells were plated in complete medium for 2 days, then medium was changed to medium without growth factors FGF and EGF. After 1 week, medium was changed to a 1:1 mixture of DMEM/F12+Glutamax and Neurobasal (Life Technologies) containing N2 (1:200) and B27 (1:100). During differentiation, half of the medium was changed every 2 to 3 days containing laminin (1:1000).

### RNA Sequencing

Total RNA was extracted using NucleoSpin RNA kit or NucleoSpin Triprep kit (Macherey-Nagel, Düren, Germany) according to the supplier’s instructions. RNA concentration was measured using Nanodrop ND-1000 and Qubit (Thermo Fisher Scientific). RNA integrity was analyzed by Bioanalyzer (Agilent Technologies, Santa Clara, CA, USA). Three biological replicates were collected for each time point in two independent experiments (except for day 14 in experiment 1). We applied the STRT (single-cell tagged reverse transcription) RNA-seq protocol [26, 27] on total bulk RNA samples with the following modifications: 10 ng of high-quality total RNA was converted into cDNA, amplified and converted to form an Illumina-compatible library. ERCC92 spike-in was used for quality control of sequenced samples and normalization of all the endogenous genes [28]. ERCC spike-in mixture was diluted 1000× with water, and 1 μl was added to reverse transcriptase cDNA master mix. In total, 25 PCR cycles were used: 15 for the first, full cDNA amplification and additional 10 to amplify and introduce sequencing-required motifs. Ready library was sequenced on three lanes of Illumina HiSeq 2000 instrument using 60 bp single reads.

RNA-seq data have been deposited in the ArrayExpress database at EMBL-EBI (https://www.ebi.ac.uk/arrayexpress) under accession number E-MTAB-7128.

### RNA-Seq Data Analysis

Data processing of the RNA-seq STRT library was performed using the STRTprep pipeline (https://github.com/shka/STRTprep/tree/v3dev [27]). Total raw reads were filtered and demultiplexed into individual samples based on the sample-specific barcodes, and redundant reads were filtered out based on UMI (unique molecular identifier) sequences. After barcodes were trimmed, processed reads were aligned to the hg19 human reference genome, synthetic ERCC92 spike-in sequences, and human ribosomal DNA unit (GenBank: U13369) using TopHat v2.0.12 [29]. Uniquely mapped reads within the 5′-UTR or 500 bp upstream of Refseq protein-coding genes were counted, and read counts were normalized by the sum of spike-in reads in each sample. Differential expression was analyzed using the R package SAMstrt [28] based on the combination of false discovery rate (FDR) < 0.01 and Benjamini-Hochberg-adjusted *p* value of the degree of variation < 0.05 [27]. The heatmap was generated using the Ward clustering method with the Spearman correlation distance matrix based on the log10-normalized expression levels of the 2516 differentially expressed genes between day 0 and day 35. The minimum value but non-zero (4.30e-05) was added to all the normalized expression levels to avoid the logarithm of zero. For the RNA-seq vs. qRT-PCR correlation plots, a constant of 0.00001 was added to all the mean values of DCX to avoid the logarithm of zero. Enrichment analysis of tissue-specific expression (UniProt UP_TISSUE) and gene ontology (GO) terms were performed using the DAVID web tool (http://david.abcc.ncifcrf.gov/) [30, 31]. GO graphs were displayed using GraphPad Prism 7 software. The gene set enrichment analysis (GSEA) was performed with GSEA v3.0 (http://software.broadinstitute.org/gsea/) using GSEAPreranked tool [32]. A total of 11,180 genes, which were expressed in at least three of the day 0 and day 35 samples, except spike-ins, were preranked according to the combination of FDR and fold change of their expression level. This gene list was then compared with the gene sets of ciliary genes and dyslexia-candidate genes. The gene set of ciliary genes was composed of 302 genes obtained from The SYSCILIA Gold Standard v1 [33]. The gene set of DCGs is shown in Suppl. Table 1. The gene set of DCGs was generated with a literature search in PubMed, including linkage and association studies and translocations and deletions co-segregating with DD, excluding CNVs (accession date: 29/5/2018). Since we chose an inclusive, exploratory approach, non-replicated genes were also included. GSEA plots were generated using ReplotGSEA.R (https://github.com/PeeperLab/Rtoolbox/blob/master/R/ReplotGSEA.R) with some modifications. For the time course analysis, 5976 genes with adjusted *p* value of the degree of variation < 0.05 were analyzed. Among them, a total of 2303 genes highly correlated between independent experiments (Pearson’s correlation coefficient > 0.7) were classified into 4 clusters, based on k-means clustering determined by X-Means algorithm using the R package Weka [34].

### Publicly Available Transcriptome Data Analysis

Two publicly available cap analysis of gene expression (CAGE) datasets were downloaded from the FANTOM (Functional annotation of the mammalian genome) 5 database (http://fantom.gsc.riken.jp/5/data/; [35]), and an RNA-seq dataset was downloaded from Gene Expression Omnibus (GEO) database [36] under the accession number GSE99469 [37]. (1) Human iPSCs to neurons by CAGE: six replicates of day 0 and six replicates of day 18 samples from 2 iPS cell lines (one from newborn male fibroblasts and another from 12-week gestation female fibroblasts) were analyzed. Promoters with over 3 counts per million (cpm) in at least 6 samples were selected. (2) Human ESCs (hESCs) to cardiomyocytes by CAGE: three replicates of day 0 and three replicates of day 12 samples from hESCs were analyzed. Promoters with over 3 cpm in at least 3 samples were selected. (3) Human iPSCs to kidney organoids by RNA-seq: three replicates of day 0 and six replicates of day 18 samples from a healthy female human iPSC line were analyzed. Genes with over 3 cpm in at least 3 samples were selected.

The read counts were normalized using the RLE method, and significantly upregulated genes or promoters were identified using the R (version 3.6.0) package “edgeR” (version 3.26.5) based on Benjamini-Hochberg-adjusted *p* value < 0.01 along with the log2-fold change > 1 in the differentiated samples against the day 0 samples. Significantly upregulated genes or official gene symbols extracted from the significantly upregulated promoters were then annotated with DAVID web tool (http://david.abcc.ncifcrf.gov/) for the GO term enrichment analysis. GO graphs were displayed using GraphPad Prism 7 software.

### Quantitative Real-Time PCR

cDNA was synthesized with Maxima First Strand cDNA Synthesis Kit (Thermo Fisher Scientific) using 500 ng of RNA. cDNA was diluted 1:5 and 2 μl of diluted cDNA was used per reaction. The analysis was performed on a 7500 Fast Real-Time PCR system (Applied Biosystems, Thermo Fisher Scientific). The reagents used for RT-qPCR were TaqMan fast Universal PCR Master Mix (Thermo Fisher Scientific) and Taqman probes (BBS2: Hs00230400_m1; DCDC2: Hs00393203_m1; DYX1C1: Hs00370049_m1; GAPDH: Hs02758991_g1; IFT57 (ESRRBL1): Hs00215973_m1; KIAA0319: Hs00207788_m1; TCTN2: Hs00430714_m1; TMEM231: Hs00226008_m1; TUBA1A: Hs00362387_m1) or FastStart Universal SYBR Green Master (Roche Diagnostics, Mannheim, Germany) and SYBR green primers (DCX_F: TTG CTG GCT GAC CTG ACG CG; DCX_R: GCT GCT AGC CAA GGA CTG GGG; GAPDH_F: CCA CAT CGC TCA GAC ACC AT; GAPDH_R: GCG CCC AAT ACG ACC AAA T; MAP2_F: AGG CAG AGA CAC AGG TGC TT; MAP2_R: GGG TTT GCT CCT AGG GTT TC; TUBB3_F: CCT ACT GCA TCG ACA ACG AG; TUBB3_R: CGA TAC CAG GTG GTT GAG GT). *GAPDH* was used as a control to normalize expression levels [21, 24]. Expression was compared relative to day 0 using the ΔΔCt method and relative expression levels were displayed as 2^^- ΔΔCt^ [38]. The data were analyzed and displayed using Microsoft Excel and GraphPad Prism 7 softwares.

### Immunocytochemistry

Immunocytochemistry was performed as described before [9, 25]. Cells were grown on glass coverslips and differentiated during 0, 7, 14, 21, 28, or 35 days as described above. Cells were fixed either with ice-cold methanol after 45 min incubation on ice or with 4% formaldehyde (Sigma-Aldrich) at room temperature, permeabilized and blocked with 0.05% PBST with 5% horse serum. Samples were incubated with primary antibody (mouse anti-acetylated alpha-tubulin, 1:5000, T7451, Sigma-Aldrich, RRID:AB_609894; rabbit anti-PCNT, 1:1000, HPA019887, Atlas antibodies, RRID: AB_1855080) overnight at 4 °C and with secondary antibody (donkey anti-rabbit Alexa 488, 1:1000, Thermo Fisher Scientific, RRID:AB_2535792; donkey anti-mouse Alexa 568, 1:1000, Thermo Fisher Scientific, RRID:AB_2534013) for 1 h at room temperature, then counterstained with DRAQ5 (1:1000, Cell Signaling Technology, Cambridge, UK) for 10 min at room temperature or with DAPI (1:1000, Sigma-Aldrich) for 1 min at room temperature. Samples were embedded in Prolong Gold antifade reagent (Thermo Fisher Scientific). Images were acquired with a Nikon A1R Ti confocal (Nikon Instruments, Inc., Melville, NY, USA) with a Plan Apo λ 60 × NA 1.4 objective with z-stack imaging mode. Images were processed using Nikon NIS-elements version 4.51 (Laboratory Imaging/Nikon), DRAQ5 was pseudocolored in blue, LUT were applied, and z-stacks were collapsed to maximum intensity projections. Images were converted to 8-bit RGB and subsequently arranged using Adobe Illustrator CS6.

## Results

### NES Cell Differentiation, Quality Control, and Global Transcriptomics Analysis

**Fig. 1:**
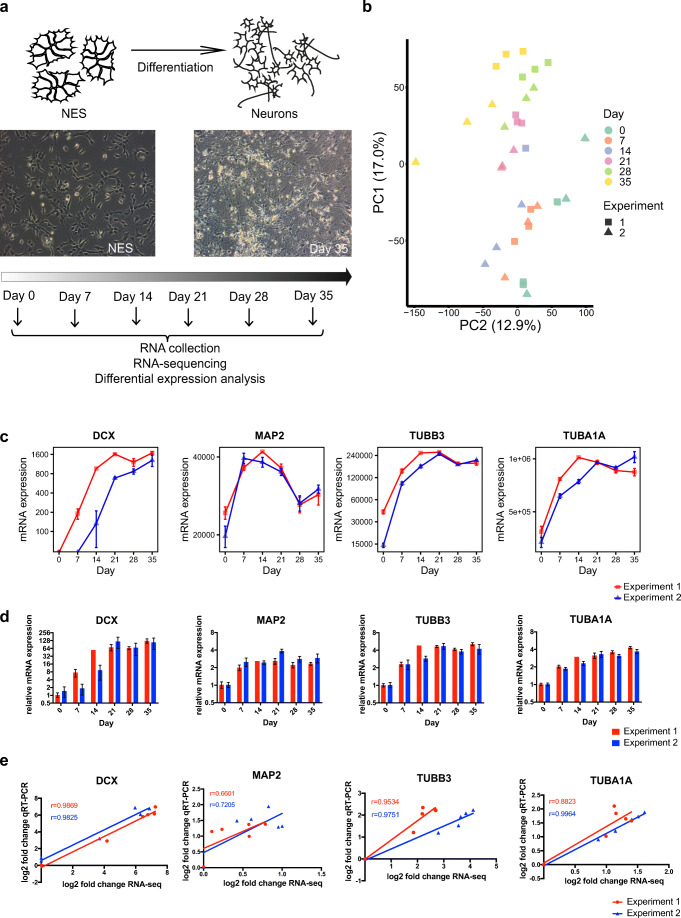
Neuronal differentiation and quality control. **a** Two independent batches (Experiment 1 + Experiment 2) of NES cells were differentiated for 35 days, and RNA samples were collected at 0, 7, 14, 21, 28, and 35 days. **b** Principal component analysis of all the samples analyzed by RNA-seq. Dot colors represent differentiation days and dot shapes represent independent experiments. PC1: principal component 1, PC2: principal component 2. **c** Time course expression pattern of the neuronal markers DCX, MAP2, TUBB3, and TUBA1A measured by RNA-seq (red: Experiment1, blue: Experiment 2). Number of molecules calculated by multiplying the normalized read counts and spike-ins are shown. Mean ± SEM are displayed. **d** qRT-PCR showing upregulation of neuronal markers DCX, MAP2, TUBB3, and TUBA1A during differentiation. Mean ± SEM are displayed, red: Experiment 1, blue: Experiment 2. **e** Correlation plots of log_2_ fold change in RNA-seq vs. log_2_ fold change in qRT-PCR. r denotes Pearson correlation coefficient

In order to model early human neuronal development, we used established protocols for NES cells (cell line AF22) [21]. We differentiated NES cells in two independent experiments towards neuronal cells during 35 days by undirected differentiation via removal of the growth factors EGF and FGF. To monitor gene expression at different stages of development, we sampled RNA at 0, 7, 14, 21, 28, and 35 days using three separately cultured replicates at each timepoint (Fig. 1a). We applied STRT RNA-seq on bulk RNA samples as a transcriptomics approach. On average, we obtained 3.7 million reads per sample, 90.6% of which were mapped to the human genome hg19 (Suppl. Table 2). First, we asked how similar the samples were according to their timepoints and experiment numbers to assess the robustness of our differentiation protocol. Sample classification by principal component analysis (PCA) of all detected genes showed that samples clustered together according to timepoints and across the two differentiation experiments (Fig. 1b).

To monitor neuronal differentiation, we tested the expression of neuronal markers. Indeed, we observed an upregulation of the established neuronal markers *DCX*, *MAP2*, *TUBB3* [39], and *TUBA1A* (Fig. 1c). We confirmed the significant upregulation of these marker genes by using qRT-PCR (Fig. 1d, Suppl. Fig. 2). Comparison of the results revealed a high concordance between RNA-seq and qRT-PCR results (Fig. 1e). In summary, these results show that the experiment was reproducible and confirm the successful differentiation of NES cells to neuronal cells.

### Characterization of Differentially Expressed Genes During Neuronal Differentiation

**Fig. 2:**
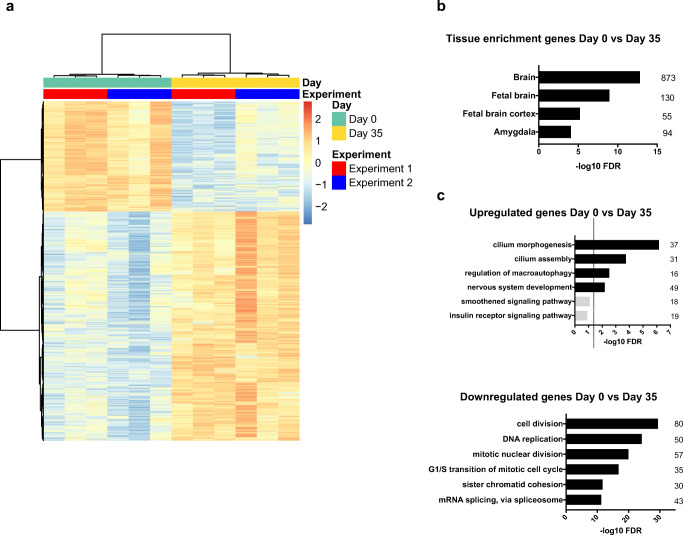
Differential gene expression analysis between day 0 and day 35. **a** Heatmap of differentially expressed genes between day 0 and day 35. All the genes at FDR < 0.01 are shown (817 downregulated, 1699 upregulated). **b** Enrichment analysis of the upregulated genes on UniProt tissue expression categories (UP_TISSUE). **c** Gene ontology term enrichment analysis of differentially expressed genes comparing day 0 and day 35. GO terms of six top ranked biological processes are shown. Terms with FDR < 0.05 are displayed in black and terms with FDR > 0.05 are displayed in gray. The dashed line indicates the limit of FDR = 0.05. The number of genes in each category is indicated on the right of the bars. *FDR* false discovery rate

Next, we asked which genes were expressed in NES cells and which genes were differentially regulated by the maturation from NES cells to neurons. Based on the results of PCA analysis, we performed differential expression analysis between the extreme timepoints day 0 and day 35 to compare the most different expression profiles. We observed that 817 genes were significantly downregulated and 1699 genes were significantly upregulated between day 0 and day 35. Unsupervised hierarchical clustering revealed that day 0 samples clustered separately from day 35 samples and that samples clustered together depending on their respective experiment number (Fig. 2a). Enrichment analysis of the 1699 upregulated genes using UniProt tissue expression categories (UP_TISSUE) revealed that 873 genes overlapped with the brain category, 130 genes with the fetal brain category, 55 genes with the fetal brain cortex category, and 94 genes with the amygdala category, confirming the neuronal identity of the differentiated cell samples (Fig. 2b).

To explore the function of the differentially regulated genes, we performed GO term enrichment analysis using the DAVID web tool [30, 31]. The six most significantly upregulated and downregulated GO terms are shown in Fig. 2c. As expected, we found neuronal development–related terms in the upregulated category and cell cycle–related terms in the downregulated category. Interestingly, we found cilia-related terms as the most significantly enriched upregulated terms (Fig. 2c). To make sure that the enrichment of ciliary genes is not a false positive signal due to a high number of tubulin genes upregulated in neurons, we examined the genes enriched in the cilium morphogenesis and cilium assembly categories. None of the genes in these categories were tubulin genes (Suppl. Table 3).

We then asked if the upregulation of cilia-related terms could also be observed in other neuronal cell lines. We performed GO analysis of the upregulated genes of iPSCs differentiated to neurons during 18 days using a publicly available cap analysis of gene expression (CAGE) dataset [35]. Indeed, we observed a strong enrichment of cilia-related GO terms, along with neuronal development-related GO terms (Suppl. Fig. 1a). Next, we asked if cilia gene upregulation can be observed as a general phenomenon in all cell types differentiating from PSCs and exiting the cell cycle. We therefore analyzed datasets from two other cell types differentiated from hESCs or hiPSCs during 12 and 18 days, respectively: cardiomyocytes [35] and kidney cells [37]. We observed some enrichment of cilia-related terms in kidney cells but not in cardiomyocytes (Suppl. Fig. 1b, c). In none of these tissues, the enrichment was as strong as for neurons. These results indicate that enrichment of cilia-related GO terms during differentiation could be specifically observed in neurons.

**Fig. 3:**
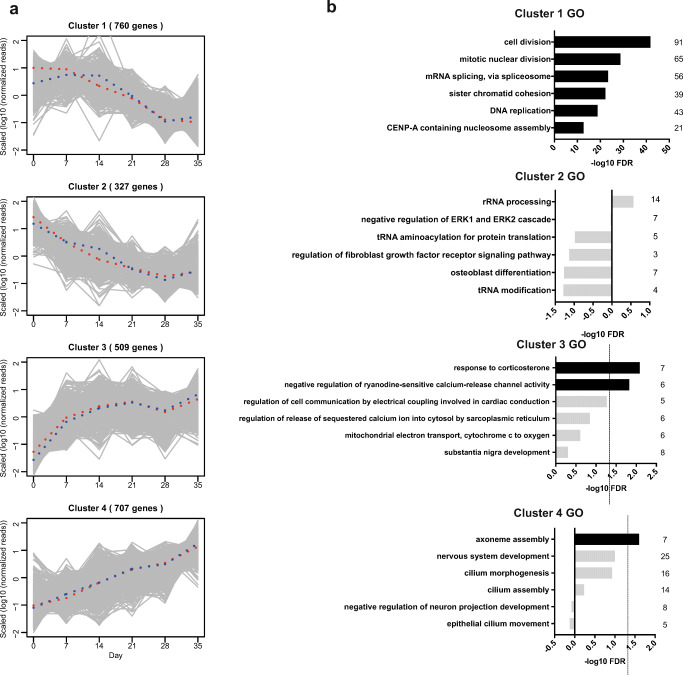
Clustering by time course gene expression analysis. **a** Clustering of all differentially expressed genes by time course expression pattern analysis resulting in clusters 1–4. **b** GO terms of biological processes associated to the clusters. GO terms of six top ranked biological processes are shown. Terms with FDR < 0.05 are displayed in black terms and terms with FDR > 0.05 are displayed in gray. The dashed line indicates the limit of FDR = 0.05. The number of genes in each category is indicated on the right of the bars. FDR false discovery rate

To further dissect the regulated gene classes, we performed clustering by time-course expression pattern analysis, revealing four different gene expression clusters (Fig. 3). Cluster 1 consisted of 760 genes, which are decreasing between day 7 and day 28 and cluster 2 comprised 327 monotonically decreasing genes. Cluster 3 contained 509 genes, which increase drastically in the beginning of differentiation, and cluster 4 consisted of 707 monotonically increasing genes (Fig. 3a). To determine the biological processes associated with the clusters, we performed GO term enrichment analysis. Cluster 1 was significantly enriched with cell cycle–related genes, further confirming the cell cycle exit of the cells after day 7 (Fig. 3b). Interestingly, cilia-related genes and nervous system development–related genes are in the same cluster (cluster 4), further indicating that they are correlated during neuronal differentiation (Fig. 3b). These results again suggest that ciliary genes might play a role during neuronal differentiation.

### Genes Associated with Ciliopathies with Neurodevelopmental Phenotypes Are Upregulated During Neuronal Differentiation

**Fig. 4:**
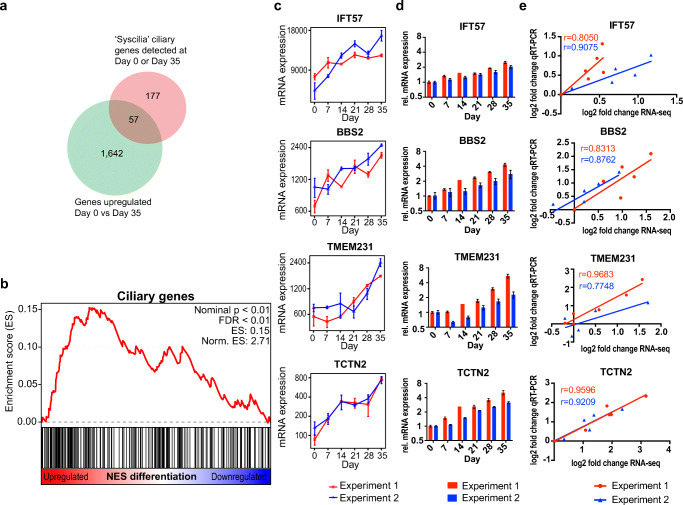
Time course expression pattern of ciliary genes. **a** Venn-diagram of all upregulated genes day 0 vs day 35 and all “Syscilia” ciliary genes detected at day 0 or 35. **b** Gene set enrichment analysis (GSEA) of “Syscilia” ciliary genes in all detected 11,180 genes. FDR false discovery rate, ES enrichment score. **c** RNA-seq time course progression plot of the ciliopathy-related genes *IFT57*, *BBS2*, *TMEM231, TCTN2*. **d** qRT-PCR showing upregulation of the dyslexia and ciliopathy-related genes *IFT57*, *BBS2*, *TMEM231, TCTN2* during neuronal differentiation. **e** Correlation plots of log_2_ fold change in RNA-seq vs. log_2_ fold change in RT-qPCR. r denotes Pearson correlation coefficient. **c**, **d** Mean ± SEM are displayed. **c**, **d**, **e** red: Experiment 1, blue: Experiment 2. *Norm.* normalized, *rel.* relative

The occurrence of significant GO terms related to cilia incited us to investigate the ciliary genes more in detail. We used a conservative list of 302 bona fide ciliary genes (The SYSCILIA Gold Standard v1 [33]); the full list of ciliary genes is provided in Suppl. Table 4. We found that 234 of those ciliary genes were expressed either at day 0 or at day 35. By comparing day 0 to day 35, we observed that 57 ciliary genes out of 234 were among the 1699 short-listed upregulated genes at day 35 showing a significant enrichment of ciliary genes (*p* value = 0.0002; Fisher’s exact test) (Fig. 4a). Similarly, GSEA analysis ranking all 11,180 detected genes and comparing them to the 234 detected ciliary genes revealed a significant enrichment (FDR < 0.01) (Fig. 4b).

Many ciliopathies display neurodevelopmental phenotypes [40]. We therefore compared all the 57 upregulated ciliary genes to the known phenotypes in ciliopathies. Of the 57 upregulated ciliary genes between day 0 and day 35, 32 genes were associated with ciliopathies of which 15 display neurodevelopmental symptoms (Suppl. Table 5). Overall, these results point to a role of ciliary genes in neuronal differentiation.

The neurodevelopmental phenotypes present in many ciliopathies [14] and the enrichment in ciliary genes observed at day 35 prompted us to characterize the time course expression pattern of examples of upregulated ciliary genes that are mutated in ciliopathies with neurocognitive phenotypes. We examined the genes *IFT57* (mutated in orofaciodigital syndrome (OFD, OMIM #311200)), *BBS2* (Bardet-Biedl syndrome (BBS, OMIM #209900)), (*TMEM231* (Meckel-Gruber syndrome (MKS, OMIM #249000)), *TCTN2* (Joubert syndrome, (JBTS, OMIM #213300)) (Fig. 4c–e). Those genes were upregulated in the RNA-seq data (Fig. 4c) and qRT-PCR data (∆∆Ct day 0 vs day 35: *p* values IFT57, BBS2, TCTN2 < 0.0001; *p* value TMEM231 < 0.001 (Student’s *t* test) (Fig. 4d; Suppl. Fig. 2)). Correlation plots revealed a high concordance between RNA-seq and qRT-PCR results (Fig. 4e). These findings indicate that ciliopathy genes producing brain phenotypes when mutated are upregulated during human neuronal differentiation in vitro, suggesting human NES cells as a good model to study ciliopathies.

### Individual Dyslexia Candidate Genes Are Upregulated During Neuronal Differentiation

**Fig. 5:**
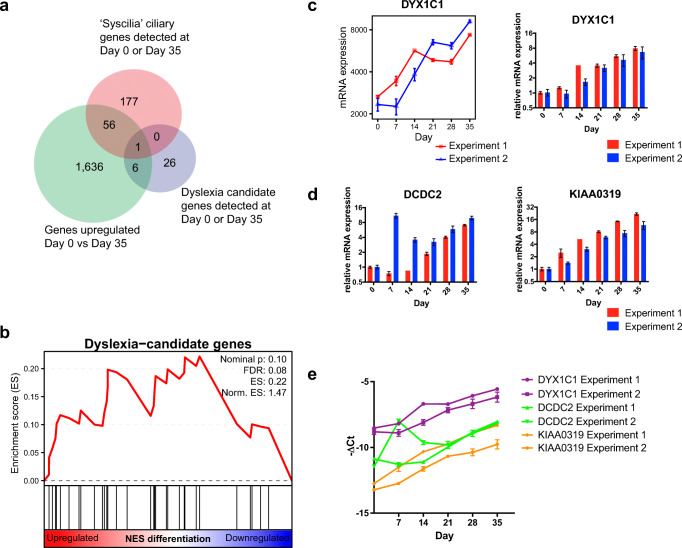
Time course expression pattern of dyslexia candidate genes. **a** Venn-diagram of all upregulated genes day 0 vs day 35, all DCGs detected at day 0 or 35 and all “Syscilia” ciliary genes detected at day 0 or 35. **b** Gene set enrichment analysis (GSEA) of DCGs in all detected 11,180 genes. FDR false discovery rate, ES enrichment score. **c***DYX1C1* expression measured by RNA-seq and qRT-PCR. **d** Relative expression of the DCGs *DCDC2* and *KIAA0319* assayed by qRT-PCR. **e** –ΔCt values by qRT-PCR of *DYX1C1*, *DCDC2*, and *KIAA0319*. **c**, **d**, **e** Mean ± SEM are displayed. **c**, **d** red: Experiment 1, blue: Experiment 2. *Norm.* normalized

Next, we addressed the expression of DCGs. A number of DCGs have been shown to have a role in neuronal development and/or in cilia prompting us to ask whether they might have a role in NES cell differentiation [41, 42]. We therefore asked whether DCGs are expressed and differentially regulated during the differentiation from NES cells to neurons. While there is an abundant literature on genomic studies on DCGs, little is known on expression and function of these genes in human neuronal development. As our approach is exploratory, we chose to use an inclusive approach towards what constitutes a DCGs rather than focusing only on replicated genes that are few. We therefore used an inclusive list of 50 DCGs containing genes identified in families with dyslexia history as well as in specific population cohorts (Suppl. Table 1). Here, we found that thirty-three of these genes were expressed either at day 0 or at day 35 or both. Of those, seven DCGs were among the 1699 short-listed genes upregulated between day 0 and day 35 (*CCDC136, COMT, DYX1C1, PRMT2, CCPG1, ZNF385D, GRIN2B*) (Fig. 5a), indicating that there was no significant enrichment overall among the short-listed upregulated genes (*p* value = 0.33, Fisher’s exact test). Similarly, GSEA analysis ranking all 11,180 detected genes and comparing them to the 33 detected DCGs showed no significant enrichment (FDR = 0.08) (Fig. 5b). These findings lead to conclude that (1) many DCGs are expressed at some point during human neuronal differentiation; however, (2) there is no significant upregulation of DCGs as a group.

Next, we examined the time course expression pattern of DCGs more in detail. For this purpose, we focused on a set of DCGs that has been highly replicated in genomic studies, namely *DYX1C1*, *DCDC2*, and *KIAA0319* [43]. Interestingly, one of the most replicated DCGs—*DYX1C1*—was one of the most highly upregulated ciliary genes (Fig. 5c), which was confirmed by qRT-PCR (Fig. 5c; Suppl. Fig. 2) (*p* value ∆∆Ct day 0 vs day 35 < 0.0001; Student’s *t* test). Expression of the two other highly replicated DCGs, *DCDC2* and *KIAA0319*, was not detected by RNA-seq, but their low-level expression was observed by qRT-PCR. Comparing their expression between day 0 and 35 by qRT-PCR revealed that they, too, were significantly upregulated during differentiation (*p* value ∆∆Ct day 0 vs day 35 < 0.0001; Student’s *t* test) (Fig. 5d; Suppl. Fig. 2). The Ct values for *DCDC2* and *KIAA0319* were much higher than the Ct values for *DYX1C1* (Fig. 5e). These results underscore the importance of *DYX1C1* in neuronal differentiation, whereas *DCDC2* and *KIAA0319* are expressed at a lower level or perhaps in specific cell types.

### Primary Cilia Are Present Throughout Differentiation of NES Cells

**Fig. 6:**
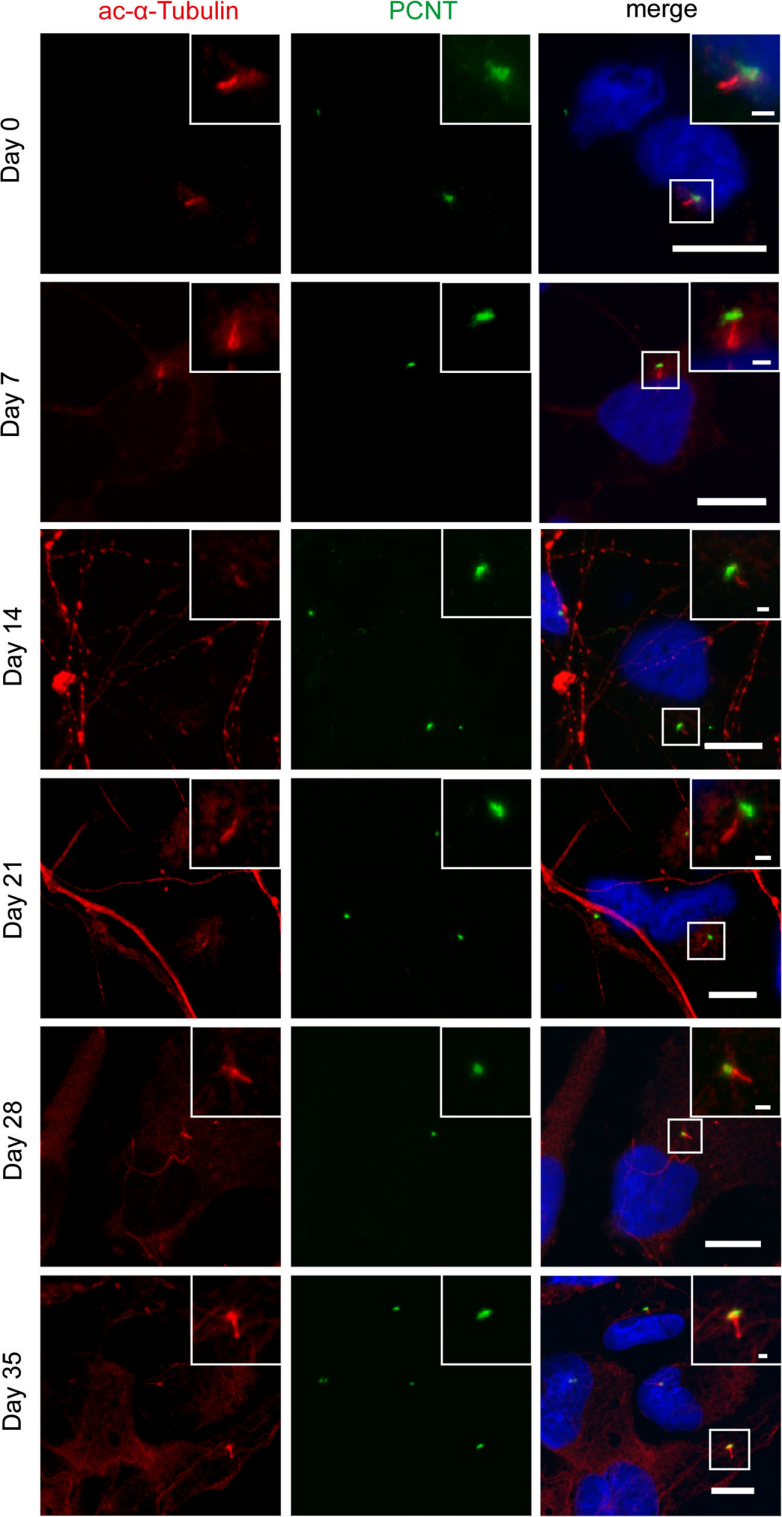
Cilia are present throughout neuronal differentiation. NES cells were plated on glass coverslips and differentiated for the indicated number of days, then fixed and stained for the ciliary marker acetylated-α-Tubulin and the centrosomal marker pericentrin (PCNT). Scale bars = 10 μm, scale bars insets = 1 μm. Nuclei were counterstained with DAPI or DRAQ5

Based on our transcriptomics results and on earlier studies describing the presence of cilia on neural progenitor cells, we asked whether primary cilia are present on the cell surface during differentiation from NES cells to neuronal cells. We therefore stained NES cells and differentiating neurons for the ciliary marker acetylated-α-tubulin [44], in combination with the centrosomal marker pericentrin (PCNT) (Fig. 6). Interestingly, cilia were present on the cell surface during all examined differentiation stages. The cilia were located on the cell body. This finding confirms that NES cells display cilia throughout development.

## Discussion

Cilia are present on the surface of most mammalian cells and have important roles in processes such as cell to cell communication [45]. However, little is known about distribution and function of primary cilia in human neuronal cells and the mechanisms by which ciliary dysfunctions lead to neurodevelopmental defects are just beginning to emerge [46]. DD is a neuropsychiatric disorder frequently associated with ciliary genes [47].

Here, we studied gene expression changes in general and of DCGs in particular during human neuronal differentiation by combining long-term self-renewing NES cells with a transcriptomics approach. Surprisingly, we found that some of the most highly upregulated GO terms were related to cilia growth. The pronounced upregulation of ciliary genes observed may be explained by more cells exiting the cell cycle, entering G0, and undergoing ciliogenesis upon removal of growth factors. However, the specific enrichment of ciliary GO terms in neurons compared with other cell types suggests that ciliary genes have a more prominent role during neuronal differentiation in particular. The upregulation of ciliary genes during human neuronal differentiation in vitro has not been widely reported yet, but Van de Leemput et al. have observed a high enrichment of cilia-related GO terms in a human ESC neuronal differentiation model consistent with our results [48]. It is known that cilia play a role in neuronal development: In early brain development, the action of pathways such as SHH, WNT, PCP, and PDGF via the primary cilium are central to brain patterning and forebrain development [13]. Signaling via the cilium also plays a role in neuronal maturation [13]. Likely, cilia regulate the balance of neurogenesis via providing access to different signaling molecules in early and later stages of neuronal development [49]. In mouse ESCs, the ciliary genes *Tmem67*, *Ahi1*, and *Ofd1* are crucial for neuronal differentiation [44, 50]. It is likely that cilia assume similar roles in the human brain.

We describe the presence of cilia on human NES and differentiating neuronal cells. This is in accordance with previous studies describing the occurrence of cilia in human neural progenitor cells (NPCs), differentiating cells in rosettes and organoids [51–53], hiPSC-derived neurons [54], and in the adult brain [55]. The *hedgehog* effector *smoothened* localizes to primary cilia in NPCs in maturing rosettes indicating that similar processes might be active in human neural cells as in rodents [51]. Cilia trigger differentiation of NPCs and might play a role in asymmetric cell division associated with neuronal differentiation [53]. The location of the cilium at the cell soma in our study is in accordance with previous observations, and it has been suggested that this localization optimizes the concentration of signaling molecules [56]. It remains to be determined whether ciliary length or number increases during differentiation.

For our study, we focused on the early neuronal development and therefore analyzed days 0 to 35. However, our data showed that the expression of most cilia genes did not yet plateau at 35 days. It would be highly interesting to analyze later timepoints, especially bearing in mind the late embryonic and postnatal growth and maturation of neuronal cilia described in rodents by Arellano and colleagues [57].

Many ciliopathies display neurodevelopmental symptoms such as mental retardation in JBTS, BBS, MKS, and OFD. More generally, 77 ciliary genes have been associated with neurodevelopmental or neurobehavioral defects [58]. In addition to their relevance in ciliopathies, ciliary genes have been associated with neuropsychiatric disorders [15–18]. One of the neurodevelopmental disorders frequently associated to ciliary genes is DD [42, 47]. It is remarkable that 33 genes out of an inclusive list of 50 DCGs were detected at day 0, day 35, or both, while seven genes were significantly upregulated during differentiation. The list of candidate genes was however not significantly enriched during neuronal differentiation using the stringent FDR cutoff of 0.05 in GSEA—yet, this does not exclude their possible roles as dyslexia candidates. As our approach was very inclusive, false-positive genes might dilute the result. It is also conceivable that these genes might function in different tissues—for example glial cells—or exert their impact on the brain in a paracrine mode. Indeed, we previously observed strong expression of *DYX1C1* in glial cells, especially astrocytes, in the FANTOM5 expression database [35]. In addition, other lines of research have pointed into the direction that glial cells might play a role in DD. For example, certain polymorphisms in the dyslexia candidate genes *DYX1C1, DCDC2*, and *KIAA0319* correlate with white matter density in the brain of children [59]. Recently, protocols to differentiate NES cells into the glial lineage have been developed, offering a way to address this question in the future [23, 60]. We further focused our analysis on a group of DCGs highly replicated in genetic studies and previously linked to neuronal development and cilia. Our results might facilitate to prioritize the candidates involved in neuronal differentiation. *DYX1C1*, the first identified DCG, has been replicated in many but not in all studies [61, 62]. It has previously been shown to act in neuronal migration [3, 63], learning, memory, and behavior [64] and later has been characterized as an axonemal dynein assembly factor [7]. Interestingly, the expression of *DYX1C1* is highly upregulated during neuronal differentiation thereby differing strongly from the expression patterns of *DNAAF1–3* that are absent or weakly expressed. These results suggest that *DYX1C1* indeed has functions other than axonemal dynein assembly, consistent with previous reports on neuronal migration and development.

*DCDC2* and *KIAA0319*—two DCGs replicated in many but not all studies—were previously associated with neuronal migration and cilia. Those genes were not detected in our RNA-seq approach, suggesting a low expression level. For *DCDC2*, this is consistent with human brain expression data from FANTOM5 and Allen Brain Atlas [35, 65]. Dcdc2 has been shown to have a role in neuronal migration and in behavior and learning [66] and localizes to rat hippocampal neuronal cilia [4, 8]. Certain variants in *DCDC2* have been associated with gray and white matter changes [59, 67, 68]. Most likely, *DCDC2* plays a role in neural development but may have a more restricted role than, e.g., *DYX1C1*, deserving further study.

Interestingly*,* while *KIAA0319* is highly expressed in developing brain tissue [5, 65], it is lowly expressed in iPSC-derived neuronal cells in vitro [35]*,* consistent with the observation in our in vitro model, implying that iPSC-derived neural cells might not be an ideal model to study *KIAA0319.* In vivo, *KIAA0319* has been implicated in neuronal migration and axon regeneration [5, 69].Whether it is involved in cilia remains unclear—it has been detected as upregulated in ciliated tissues, and it is interesting to note that its protein possesses five PKD domains [11, 70, 71].

We here described the regulation of ciliary genes and investigated the expression of DCGs in a human neuronal in vitro model. The present findings should have implications for future work on ciliopathies and DD and prompt more studies on the neurodevelopmental roles of ciliary genes in human. Due to the immediate clinical relevance, a more thorough understanding of the functions of ciliary genes in human neurons is highly relevant. While our work provides a resource using a transcriptomics approach, it also sets the stage for future studies on patient-derived iPS/NES cells and modeling of neurodevelopmental disorders connected to cilia. While we do not provide evidence for a direct causal connection of ciliary genes and DCGs, we for the first time provide a systematic approach of their expression patterns in a human neuronal in vitro model and show that human NES cells provide a valid model to study ciliopathies or DCGs. Future functional studies should address the molecular mechanisms underlying the involvement of ciliary genes and DCGs in human brain development.

## Electronic supplementary material

ESM 1 (DOC 697 kb)

## Abbreviations

BBS: Bardet-Biedl syndrome
CAGE: Cap analysis of gene expression
DAVID: Database for Annotation, Visualization and Integrated Discovery
DCDC2: Doublecortin domain containing 2
DCG: Dyslexia candidate gene
DCX: Doublecortin
DD: Developmental dyslexia
DNAAF: Dynein axonemal assembly factor
DYX1C1: Dyslexia susceptibility 1 candidate 1
EGF: Epidermal growth factor
FANTOM: Functional annotation of the mammalian genome
FDR: False discovery rate
FGF: Fibroblast growth factor
GEO: Gene Expression Omnibus
GO: Gene ontology
GPCR: G protein coupled receptor
GSEA: Gene set enrichment analysis
hESC: Human embryonic stem cell
hiPSC: Human induced pluripotent stem cell
IFT57: Intraflagellar transport 57
JBTS: Joubert syndrome
MAP2: Microtubule-associated protein 2
MKS: Meckel-Gruber syndrome
NES cells: Long-term self-renewing neuroepithelial stem cells
NPC: Neural progenitor cell
OFD: Orofaciodigital syndrome
PCA: Principal component analysis
PCNT: Pericentrin
STRT: Single cell tagged reverse transcription
TCTN2: Tectonic family member 2
TMEM231: Transmembrane protein 231
TUBA1A: Tubulin α1α
TUBB3: Tubulin β3
UMI: Unique molecular identifier
