# Supplementary material for: Dyslexia Candidate Gene and Ciliary Gene Expression Dynamics During Human Neuronal Differentiation
Source: Mol Neurobiol. 2020 May 22;57(7):2944–58. doi: 10.1007/s12035-020-01905-6 (PMC7320047; doi:10.1007/s12035-020-01905-6)
Supplement: Supplementary file 1 — (DOC 697 kb) [file 12035_2020_1905_MOESM1_ESM.doc]

***Supplementary material***

Molecular Neurobiology

**Dyslexia candidate gene and ciliary gene expression dynamics during human neuronal differentiation**

Andrea Bieder^*#^, Masahito Yoshihara^*^, Shintaro Katayama, Kaarel Krjutškov, Anna Falk, Juha Kere ^#^, Isabel Tapia-Páez

^#^corresponding authors: Andrea Bieder, Department of Biosciences and Nutrition, Karolinska Institutet, Hälsovägen 9, 141 57 Huddinge, Sweden, andrea.bieder@ki.se, 0046 73 738 13 95, and Juha Kere, Department of Biosciences and Nutrition, Karolinska Institutet, Hälsovägen 9, 141 57 Huddinge, Sweden, juha.kere@ki.se, 0046 73 421 35 50


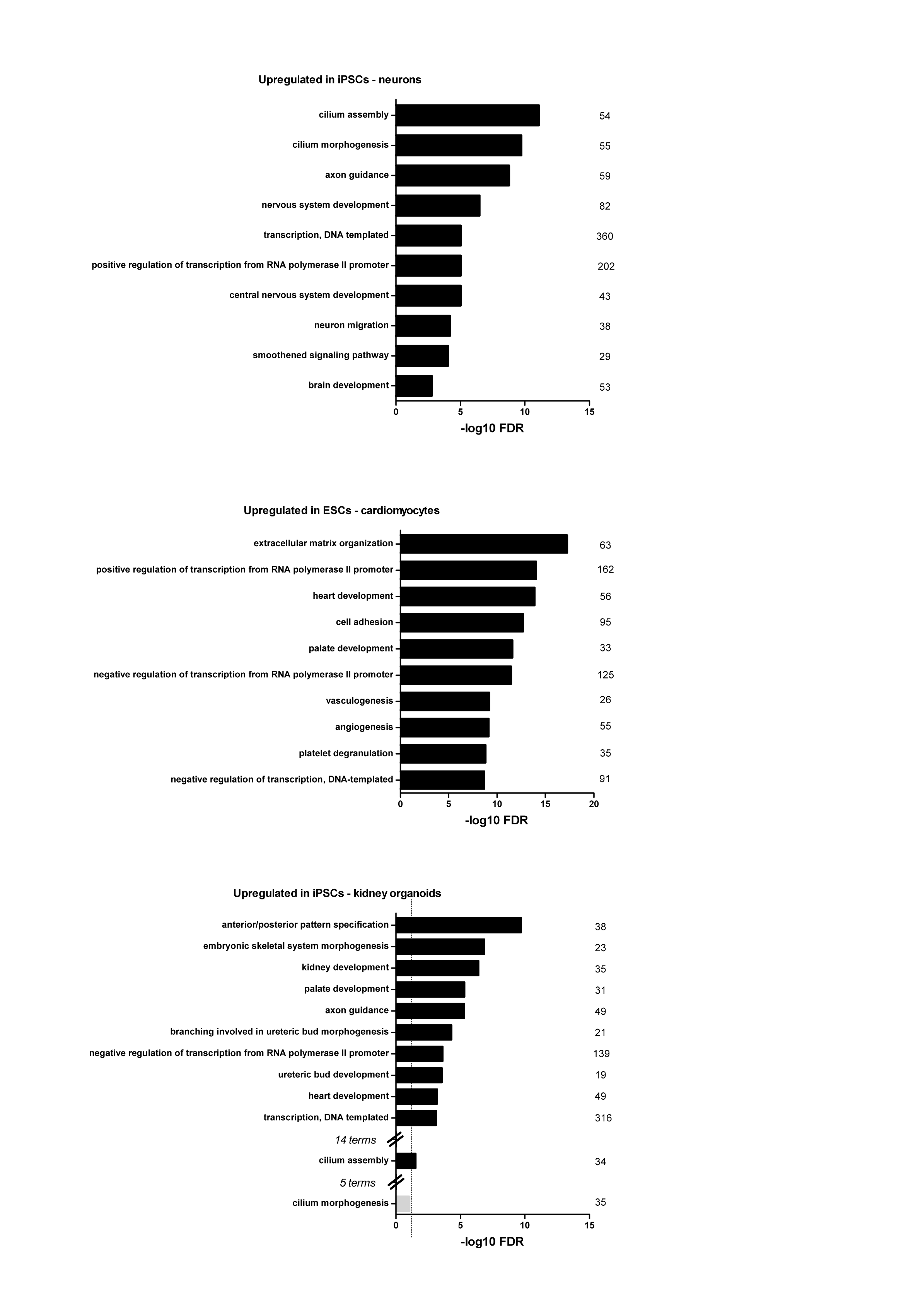


**Suppl. Fig.1: Gene ontology term enrichment analysis of upregulated genes in differentiating cell lines from various tissues.** Top ten terms and cilia-related terms are displayed. **a)** hiPSCs to neurons, day 0 vs. day 18 **b)** hESCs to cardiomyocytes, day 0 vs. day 12. No cilia-related GO terms were detected in cardiomyocytes (712 GO terms in total). **c)** hiPSCs to kidney organoids, day 0 vs. day 18. The dashed line indicates the limit of FDR=0.05. Terms with FDR<0.05 are displayed in black and terms with FDR>0.05 are displayed in gray. The number of genes in each term is indicated on the right.

**a**

**b**

**c**


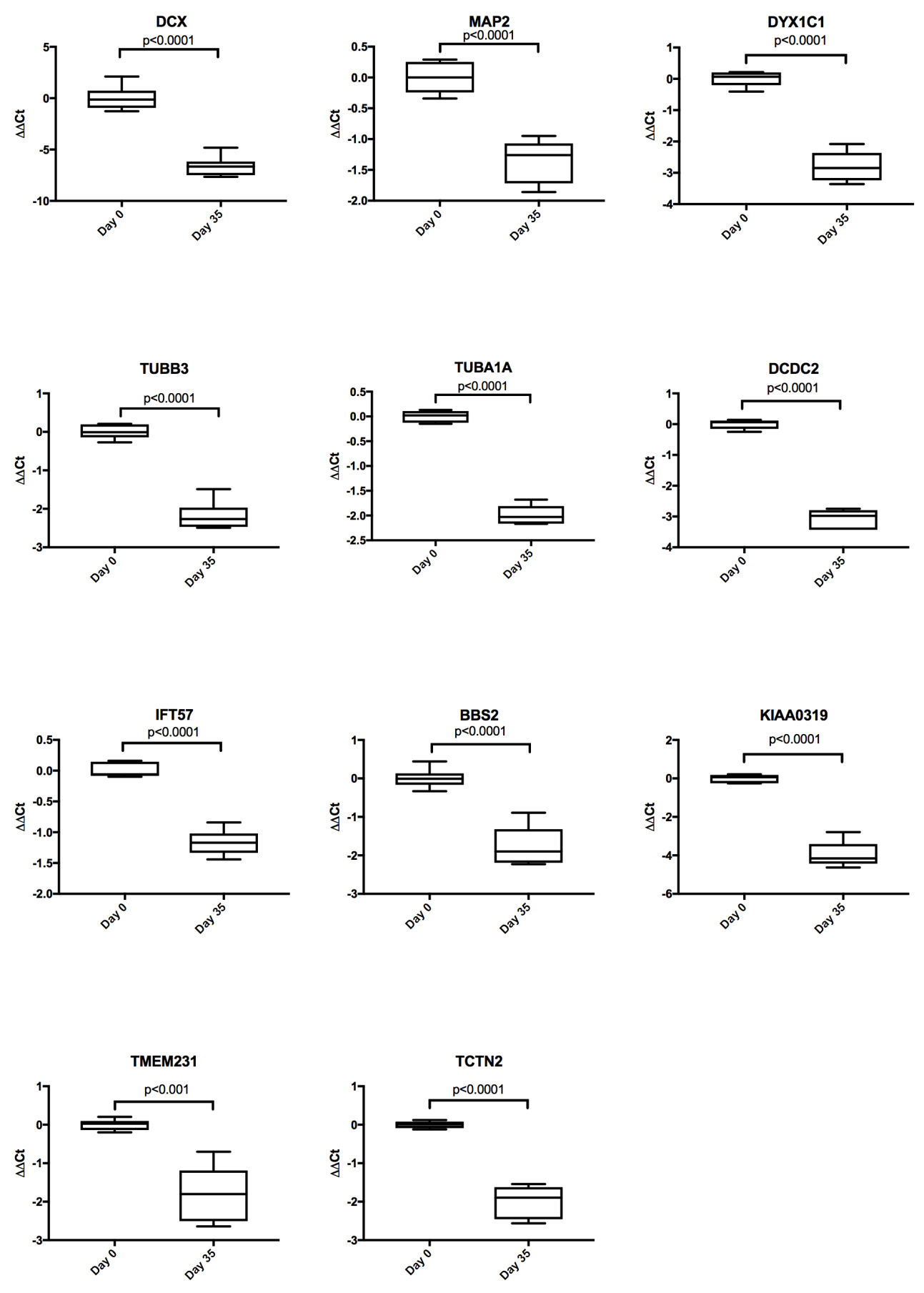


**Suppl. Fig. 2: Statistical analysis of qRT-PCR day 0 vs. day 35.** ∆∆Ct values and Student´s t-test.

| **Suppl. Table 1: Candidate genes for developmental dyslexia or reading related traits** | | | | | | | | |  | |
| --- | --- | --- | --- | --- | --- | --- | --- | --- | --- | --- |
|  | |  | | |  | | | |  | |
| **NCBI Gene name** | | **Alternative gene name** | | | **References** | | | |  | |
| ARHGAP26 | |  | | | Naskar et al., 2018 | | | |  | |
| CCDC136 | |  | | | Gialluisi et al., 2014; Adams et al., 2017 | | | |  | |
| CCPG1 | |  | | | Taipale et al., 2003 | | | |  | |
| CDK11A | | CDC2L2 | | | Luciano et al., 2013 | | | |  | |
| CDK11B | | CDC2L1 | | | Luciano et al., 2013 | | | |  | |
| CEP63 | |  | | | Einarsdottir et al., 2015 | | | |  | |
| CMIP | |  | | | Newbury et al., 2011; Scerri et al., 2011 | | | |  | |
| CNTNAP2 | |  | | | Newbury et al., 2011; Peter et al., 2011 | | | |  | |
| COMT | |  | | | Landi et al., 2013 | | | |  | |
| CSMD1 | |  | | | Einarsdottir et al., 2017 | | | |  | |
| CTNND2 | |  | | | Hofmeister et al., 2015 | | | |  | |
| CYP19A1 | |  | | | Anthoni et al., 2012 | | | |  | |
| DCDC2 | |  | | | Meng et al., 2005b | | | |  | |
| DGKI | |  | | | Matsson et al., 2011 | | | |  | |
| DIP2A | |  | | | Poelmans et al., 2009; Kong et al., 2016 | | | |  | |
| DNAAF4 | | DYX1C1 | | | Taipale et al., 2003 | | | |  | |
| DOCK4 | |  | | | Pagnamenta et al., 2010 | | | |  | |
| DRD2 | |  | | | Chen et al., 2014 | | | |  | |
| DRD4 | |  | | | Hsiung et al., 2004 | | | |  | |
| DYM | |  | | | Scerri et al., 2010 | | | |  | |
| FLNC | |  | | | Gialluisi et al., 2014; Adams et al., 2017 | | | |  | |
| FOXP2 | |  | | | Peter et al., 2011 | | | |  | |
| GCFC2 | | C2ORF3 | | | Anthoni et al., 2007; Scerri et al., 2012 | | | |  | |
| GNPTAB | |  | | | Chen et al., 2015 | | | |  | |
| GRIN2B | |  | | | Ludwig et al., 2010; Mascheretti et al., 2015 | | | |  | |
| HRAS | |  | | | Hsiung et al., 2004 | | | |  | |
| KIAA0319 | |  | | | Cope et al., 2005 | | | |  | |
| KIAA0319L | |  | | | Couto et al., 2008 | | | |  | |
| MC5R | |  | | | Scerri et al., 2010 | | | |  | |
| MRPL19 | |  | | | Anthoni et al., 2007; Scerri et al., 2012 | | | |  | |
| MYO5B | |  | | | Mueller et al., 2014 | | | |  | |
| NAGPA | |  | | | Chen et al., 2015 | | | |  | |
| NCAN | |  | | | Einarsdottir et al., 2017 | | | |  | |
| NEDD4L | |  | | | Scerri et al., 2010, Mueller et al., 2014 | | | |  | |
| NOP9 | |  | | | Pettigrew et al., 2016 | | | |  | |
| PCDH1 | |  | | | Naskar et al., 2018 | | | |  | |
| PCNT | |  | | | Poelmans et al., 2009 | | | |  | |
| PDE7B | |  | | | Buonincontri et al., 2011 | | | |  | |
| PRMT2 | |  | | | Poelmans et al., 2009 | | | |  | |
| RBFOX2 | |  | | | Gialluisi et al., 2014 | | | |  | |
| RCAN3 | |  | | | Luciano et al., 2013 | | | |  | |
| ROBO1 | |  | | | Hannula-Jouppi et al., 20005 | | | |  | |
| S100B | |  | | | Poelmans et al., 2009; Matsson et al., 2015 | | | |  | |
| SLC35E2B | | LOC728661 | | | Luciano et al., 2013 | | | |  | |
| TAF7 | |  | | | Naskar et al., 2018 | | | |  | |
| TCF12 | |  | | | Buonincontri et al., 2011 | | | |  | |
| TDP2 | | TTRAP | | | Luciano et al., 2007 | | | |  | |
| ZNF280D | |  | | | Buonincontri et al., 2011 | | | |  | |
| ZNF385D | |  | | | Eicher et al., 2013 | | | |  | |
| ZNF804A | |  | | | Becker et al., 2012 | | | |  | |
| **Suppl. Table 2: Sequence summary table** | | | |  | |  |  |  | |  |
|  |  | |  |  | |  |  |  | |  |
| **WELL** | **NAME** | | **Experiment** | **Day** | | **Replicate** | **Total reads** | **Mapped reads** | | **Mapped rate** |
| A1 | Ex1-Day0-1 | | 1 | 0 | | 1 | 5176437 | 4700728 | | 90,8% |
| A2 | Ex1-Day0-2 | | 1 | 0 | | 2 | 3245721 | 2944136 | | 90,7% |
| A3 | Ex1-Day0-3 | | 1 | 0 | | 3 | 4223091 | 3847049 | | 91,1% |
| A4 | Ex1-Day7-1 | | 1 | 7 | | 1 | 4685369 | 4239235 | | 90,5% |
| A5 | Ex1-Day7-2 | | 1 | 7 | | 2 | 4019516 | 3656047 | | 91,0% |
| A6 | Ex1-Day7-3 | | 1 | 7 | | 3 | 4011207 | 3632665 | | 90,6% |
| A7 | Ex1-Day14-1 | | 1 | 14 | | 1 | 3865659 | 3504117 | | 90,6% |
| A8 | Ex1-Day21-1 | | 1 | 21 | | 1 | 3427668 | 3098530 | | 90,4% |
| B1 | Ex1-Day21-2 | | 1 | 21 | | 2 | 4140821 | 3737985 | | 90,3% |
| B2 | Ex1-Day21-3 | | 1 | 21 | | 3 | 4287090 | 3860186 | | 90,0% |
| B3 | Ex1-Day28-1 | | 1 | 28 | | 1 | 2460692 | 2253134 | | 91,6% |
| B4 | Ex1-Day28-2 | | 1 | 28 | | 2 | 3230580 | 2927461 | | 90,6% |
| B5 | Ex1-Day28-3 | | 1 | 28 | | 3 | 2415401 | 2201105 | | 91,1% |
| B6 | Ex1-Day35-1 | | 1 | 35 | | 1 | 3623947 | 3278280 | | 90,5% |
| B7 | Ex1-Day35-2 | | 1 | 35 | | 2 | 2898603 | 2635747 | | 90,9% |
| B8 | Ex1-Day35-3 | | 1 | 35 | | 3 | 3042900 | 2752114 | | 90,4% |
| F1 | Ex2-Day0-1 | | 2 | 0 | | 1 | 3245876 | 2930677 | | 90,3% |
| C2 | Ex2-Day0-2 | | 2 | 0 | | 2 | 4607577 | 4108361 | | 89,2% |
| C3 | Ex2-Day0-3 | | 2 | 0 | | 3 | 4170304 | 3786866 | | 90,8% |
| C7 | Ex2-Day7-1 | | 2 | 7 | | 1 | 3539726 | 3221853 | | 91,0% |
| C8 | Ex2-Day7-2 | | 2 | 7 | | 2 | 3361497 | 3073150 | | 91,4% |
| D1 | Ex2-Day7-3 | | 2 | 7 | | 3 | 3580105 | 3223810 | | 90,0% |
| D2 | Ex2-Day14-1 | | 2 | 14 | | 1 | 4924660 | 4472631 | | 90,8% |
| D3 | Ex2-Day14-2 | | 2 | 14 | | 2 | 3339932 | 3005396 | | 90,0% |
| D4 | Ex2-Day14-3 | | 2 | 14 | | 3 | 5163626 | 4667676 | | 90,4% |
| D5 | Ex2-Day21-1 | | 2 | 21 | | 1 | 3587157 | 3247192 | | 90,5% |
| D6 | Ex2-Day21-2 | | 2 | 21 | | 2 | 4182931 | 3781549 | | 90,4% |
| D7 | Ex2-Day21-3 | | 2 | 21 | | 3 | 4095059 | 3719445 | | 90,8% |
| D8 | Ex2-Day28-1 | | 2 | 28 | | 1 | 3234940 | 2907271 | | 89,9% |
| E1 | Ex2-Day28-2 | | 2 | 28 | | 2 | 3396812 | 3115056 | | 91,7% |
| E2 | Ex2-Day28-3 | | 2 | 28 | | 3 | 4675350 | 4218262 | | 90,2% |
| E3 | Ex2-Day35-1 | | 2 | 35 | | 1 | 4063148 | 3671626 | | 90,4% |
| E4 | Ex2-Day35-2 | | 2 | 35 | | 2 | 3427562 | 3115704 | | 90,9% |
| E5 | Ex2-Day35-3 | | 2 | 35 | | 3 | 3025931 | 2745237 | | 90,7% |

| **Suppl. Table 3: Enriched genes in cilia-related GO categories** | |
| --- | --- |
|  |  |
| **GO:0060271~cilium morphogenesis** | **GO:0042384~cilium assembly** |
| BBS4 | CLUAP1 |
| SEPT2 | BBS4 |
| B9D1 | SEPT2 |
| CFAP221 | B9D1 |
| TTC8 | TTC8 |
| TMEM17 | TMEM17 |
| SPAG16 | SPAG16 |
| TROVE2 | CFAP53 |
| TMEM231 | TMEM231 |
| BBIP1 | BBIP1 |
| IFT46 | IFT46 |
| IFT20 | IFT20 |
| GSN | CEP290 |
| TCTN3 | TCTN2 |
| CEP290 | SSX2IP |
| TCTN1 | TMEM107 |
| TCTN2 | ATP6V0D1 |
| SSX2IP | CCDC28B |
| IFT43 | RPGR |
| ATP6V0D1 | KIF3A |
| TMEM107 | CBY1 |
| CCDC28B | BBS1 |
| RPGR | NME5 |
| KIF3A | BBS2 |
| WWTR1 | FNBP1L |
| ARL3 | IFT57 |
| BBS1 | CCDC113 |
| BBS2 | CC2D2A |
| C21ORF2 | IFT81 |
| IFT57 | RFX2 |
| CCDC113 | SNX10 |
| CC2D2A |  |
| IFT81 |  |
| RFX2 |  |
| SEPT7 |  |
| IFT88 |  |
| SNX10 |  |

| **Suppl. Table 4: Syscilia Gold Standard gene list (van Dam et al., 2013)** | | | | | | | |  |  |  |
| --- | --- | --- | --- | --- | --- | --- | --- | --- | --- | --- |
|  |  |  |  |  |  |  |  |  |  |  |
| **Gene name** | **up Day 0 vs. Day 35** |  | **Gene name** | **up Day 0 vs. Day 35** |  | **Gene name** | **up Day 0 vs. Day 35** |  | **Gene name** | **up Day 0 vs. Day 35** |
| ADCY3 |  |  | CLUAP1 | Yes |  | GLI1 |  |  | MDM1 |  |
| AHI1 |  |  | CNGA2 |  |  | GLI2 |  |  | MKKS |  |
| AK7 |  |  | CNGA4 |  |  | GLI3 |  |  | MKS1 |  |
| AK8 |  |  | CNGB1 |  |  | GLIS2 |  |  | MLF1 | Yes |
| ALMS1 |  |  | CP110 |  |  | GPR161 |  |  | MNS1 |  |
| ARF4 | Yes |  | CRB3 |  |  | GPR98 |  |  | MYO15A |  |
| ARL13B |  |  | CROCC |  |  | GSK3B |  |  | MYO7A |  |
| ARL3 | Yes |  | CTNNB1 |  |  | HAP1 |  |  | NEK1 |  |
| ARL6 |  |  | DCDC2 |  |  | HEATR2 |  |  | NEK2 |  |
| ASAP1 |  |  | DFNB31 |  |  | HNF1B |  |  | NEK4 | Yes |
| ATXN10 |  |  | DISC1 |  |  | HSPA8 |  |  | NEK8 |  |
| AZI1 |  |  | DNAAF1 |  |  | HSPB11 |  |  | NGFR |  |
| B9D1 | Yes |  | DNAAF2 |  |  | HTR6 |  |  | NIN |  |
| B9D2 |  |  | DNAAF3 |  |  | HTT |  |  | NINL |  |
| BBS1 | Yes |  | DNAH1 |  |  | HYDIN |  |  | NME5 | Yes |
| BBS10 |  |  | DNAH10 |  |  | HYLS1 |  |  | NME7 |  |
| BBS12 |  |  | DNAH11 |  |  | IFT122 |  |  | NME8 |  |
| BBS2 | Yes |  | DNAH2 |  |  | IFT140 |  |  | NOTO |  |
| BBS4 | Yes |  | DNAH5 |  |  | IFT172 |  |  | NPHP1 | Yes |
| BBS5 |  |  | DNAH6 |  |  | IFT20 | Yes |  | NPHP3 |  |
| BBS7 |  |  | DNAI1 |  |  | IFT27 |  |  | NPHP4 |  |
| BBS9 |  |  | DNAI2 |  |  | IFT43 | Yes |  | NUP214 |  |
| C21orf2 | Yes |  | DNAL1 | Yes |  | IFT46 | Yes |  | NUP35 |  |
| C2CD3 |  |  | DNALI1 | Yes |  | IFT52 |  |  | NUP37 |  |
| C2orf71 |  |  | DPCD | Yes |  | IFT57 | Yes |  | NUP62 |  |
| C8orf37 | Yes |  | DPYSL2 |  |  | IFT74 |  |  | NUP93 |  |
| CBY1 | Yes |  | DRD1 |  |  | IFT80 |  |  | OCRL |  |
| CC2D2A | Yes |  | DRD2 |  |  | IFT81 | Yes |  | ODF2 |  |
| CCDC103 | Yes |  | DRD5 |  |  | IFT88 | Yes |  | OFD1 |  |
| CCDC114 |  |  | DVL1 |  |  | INPP5E |  |  | ORC1 |  |
| CCDC164/DRC1 |  |  | DYNC2H1 |  |  | INTU |  |  | PACRG | Yes |
| CCDC28B | Yes |  | DYNLT1 | Yes |  | INVS |  |  | PAFAH1B1 |  |
| CCDC37 |  |  | DYX1C1/DNAAF4 | Yes |  | IQCB1 |  |  | PARD3 |  |
| CCDC39 |  |  | EFHC1 |  |  | KIF17 |  |  | PARD6A |  |
| CCDC40 |  |  | EVC |  |  | KIF19 |  |  | PCDH15 |  |
| CCDC41 |  |  | EVC2 |  |  | KIF24 |  |  | PCM1 |  |
| CDH23 |  |  | EXOC3 |  |  | KIF27 |  |  | PDE6D | Yes |
| CENPJ |  |  | EXOC4 |  |  | KIF3A | Yes |  | PDZD7 |  |
| CEP104 |  |  | EXOC5 |  |  | KIF3B |  |  | PHF17 |  |
| CEP135 |  |  | EXOC6 |  |  | KIF3C |  |  | PIBF1 |  |
| CEP164 |  |  | EXOC6B |  |  | KIF7 |  |  | PKD1 |  |
| CEP250 |  |  | FAM161A |  |  | LCA5 |  |  | PKD1L1 |  |
| CEP290 | Yes |  | FBF1 |  |  | LRRC6 | Yes |  | PKD2 |  |
| CEP41 |  |  | FLNA |  |  | LZTFL1 |  |  | PKHD1 |  |
| CEP72 |  |  | FOPNL |  |  | MAK |  |  | PLK1 |  |
| CEP89 |  |  | FOXJ1 |  |  | MAL | Yes |  | POC1A |  |
| CEP97 |  |  | FUZ |  |  | MAPRE1 |  |  | PTCH1 |  |
| CLDN2 |  |  | GAS8 |  |  | MCHR1 |  |  | PTPDC1 |  |

|  |  |  |  |  |  |  |  |
| --- | --- | --- | --- | --- | --- | --- | --- |
|  |  |  |  |  |  |  |  |
| **Gene name** | **up Day 0 vs. Day 35** |  | **Gene name** | **up Day 0 vs. Day 35** |  | **Gene name** | **up Day 0 vs. Day 35** |
| RAB11A |  |  | SYNE2 |  |  | TULP3 |  |
| RAB11FIP3 |  |  | TBC1D30 |  |  | ULK4 |  |
| RAB17 |  |  | TBC1D7 |  |  | USH1C |  |
| RAB23 |  |  | TCTN1 | Yes |  | USH1G |  |
| RAB3IP |  |  | TCTN2 | Yes |  | USH2A |  |
| RAB8A |  |  | TCTN3 | Yes |  | VDAC3 |  |
| RABL5/IFT22 | Yes |  | TEKT2 | Yes |  | VHL |  |
| RAN |  |  | TEKT4 |  |  | WDPCP |  |
| RANBP1 |  |  | TEKT5 |  |  | WDR19 |  |
| RFX3 |  |  | TMEM138 |  |  | WDR35 |  |
| RILPL1 |  |  | TMEM216 |  |  | WDR60 |  |
| RILPL2 |  |  | TMEM231 | Yes |  | WDR78 |  |
| ROPN1L |  |  | TMEM237 |  |  | XPNPEP3 |  |
| RP1 |  |  | TMEM67 |  |  | ZNF423 |  |
| RP2 |  |  | TNPO1 |  |  |  |  |
| RPGR | Yes |  | TOPORS |  |  |  |  |
| RPGRIP1 |  |  | TPPP2 |  |  |  |  |
| RPGRIP1L |  |  | TRAF3IP1 |  |  |  |  |
| RSPH1 | Yes |  | TRAPPC10 |  |  |  |  |
| RSPH3 |  |  | TRAPPC3 | Yes |  |  |  |
| RSPH4A |  |  | TRAPPC9 |  |  |  |  |
| RSPH9 |  |  | TRIM32 |  |  |  |  |
| RTTN |  |  | TRIP11 |  |  |  |  |
| SASS6 |  |  | TTBK2 |  |  |  |  |
| SCLT1 |  |  | TTC12 |  |  |  |  |
| SDCCAG8 | Yes |  | TTC21B |  |  |  |  |
| SEPT2 | Yes |  | TTC26 |  |  |  |  |
| SEPT7 | Yes |  | TTC29 |  |  |  |  |
| SGK196 |  |  | TTC30A |  |  |  |  |
| SHH |  |  | TTC30B |  |  |  |  |
| SLC47A2 |  |  | TTC8 | Yes |  |  |  |
| SMO |  |  | TTK |  |  |  |  |
| SNAP25 | Yes |  | TTLL3 |  |  |  |  |
| SNX10 | Yes |  | TTLL6 |  |  |  |  |
| SPA17 | Yes |  | TTLL9 |  |  |  |  |
| SPAG16 | Yes |  | TUBA1A | Yes |  |  |  |
| SPAG17 |  |  | TUBA1C |  |  |  |  |
| SPAG6 |  |  | TUBA4A |  |  |  |  |
| SPATA7 | Yes |  | TUBB2A | Yes |  |  |  |
| SPEF2 |  |  | TUBB2B | Yes |  |  |  |
| SSNA1 |  |  | TUBB3 | Yes |  |  |  |
| SSTR3 |  |  | TUBE1 |  |  |  |  |
| STIL |  |  | TUBGCP2 |  |  |  |  |
| STK36 |  |  | TUBGCP3 |  |  |  |  |
| STK38L |  |  | TUBGCP4 |  |  |  |  |
| STOML3 |  |  | TUBGCP5 |  |  |  |  |
| STX3 |  |  | TUBGCP6 |  |  |  |  |
| SUFU |  |  | TULP1 |  |  |  |  |

| **Suppl. Table 5: Upregulated 'Syscilia' ciliary genes Day 0 vs Day 35 and corresponding ciliopathy** | | | | | |
| --- | --- | --- | --- | --- | --- |
| **including brain phenotype** | |  |  |  |  |
|  |  |  |  |  |  |
| **Gene name** | **Ciliopathy** | **Brain phenotype** |  |  |  |
| ARF4 | - |  |  | Ciliopathies: |  |
| ARL3 | RP |  |  | JATD: Jeune asphyxiating thoracic dystrophy |  |
| B9D1 | MKS | OE |  | RP: retinitis pigmentosa |  |
| BBS1 | BBS | ID |  | BBS: Bardet-Biedl syndrome |  |
| BBS2 | BBS | ID |  | PCD: primary ciliary dyskinesia |  |
| BBS4 | BBS | ID |  | NPHP: Nephronophthisis |  |
| C21orf2 | JATD/RP |  |  | SLS: Senior-Loken sydnrome | |
| C8orf37 | BBS/RP | ? |  | JBTS: Joubert syndrome |  |
| CBY1 | - |  |  | MKS: Meckel-Gruber syndrome | |
| CC2D2A | JBTS/MKS | CVH, ID |  | OFD: Oro-facio-digital syndrome |  |
| CCDC103 | PCD |  |  | CED: cranioectodermal dysplasia | |
| CCDC28B | BBS |  |  | LCA: Leber congenital amaurosis | |
| CEP290 | NPHP/SLS/JBTS/MKS/BBS | CVH, CBD |  |  |  |
| CLUAP1 | JBTS/OFD |  |  | Brain phenotypes: |  |
| DNAAF4 | PCD |  |  | CVH: cerebellar vermis hypoplasia | |
| DNAL1 | PCD |  |  | CBD: congenital brain defects |  |
| DNALI1 | PCD |  |  | ID: intellectual disability |  |
| DPCD | PCD |  |  | HC: hydrocephalus | |
| DRC1 | PCD |  |  | OE: OE, occipital emphalocele | |
| DYNLT1 | - |  |  |  | |
| IFT20 | - |  |  | Reference: Braun and Hildebrandt, 2017 |  |
| IFT22 | - |  |  |  | |
| IFT43 | CED |  |  |  |  |
| IFT46 | - |  |  |  |  |
| IFT57 | OFD |  |  |  |  |
| IFT81 | IFT81? (PD) | CVH |  |  |  |
| IFT88 | - |  |  |  |  |
| KIF3A | - |  |  |  |  |
| LRRC6 | PCD |  |  |  |  |
| MAL | - |  |  |  |  |
| MLF1 | - |  |  |  | |
| NME5 | - |  |  |  | |
| NPHP1 | NPHP | CVH, rare |  |  |  |
| PACRG | - |  |  |  | |
| PDE6D | JBTS | CVH, CBD, ID |  |  |  |
| RPGR | RP |  |  |  |  |
| RSPH1 | PCD |  |  |  |  |
| SDCCAG8 | SLS/BBS | ID |  |  |  |
| SEPT2 | - |  |  |  |  |
| SEPT7 | - |  |  |  |  |
| SNAP25 | - |  |  |  |  |
| SNX10 | - |  |  |  |  |
| SPA17 | - |  |  |  |  |
| SPAG16 | - |  |  |  | |
| SPATA7 | (LCA/RP) |  |  |  |  |
| TCTN1 | JBTS | CVH |  |  | |
| TCTN2 | JBTS/MKS | CVH, ID, CBD |  |  |  |
| TCTN3 | JBTS/OFD | CVH, OE, ID |  |  |  |
| TEKT2 | - |  |  |  |  |
| TMEM231 | JBTS/OFD/MKS | CVH, ID, OE |  |  |  |
| TRAPPC3 | - |  |  |  | |
| TTC8 | BBS | ID |  |  |  |
| TUBA1A | - |  |  |  | |
| TUBB2A | - |  |  |  | |
| TUBB2B | - |  |  |  | |
| TUBB3 | - |  |  |  |  |
